# Determinants of stunting among children under age five in Burundi: Evidence from the 2016–2017 Burundi Demographic and Health Survey (BDHS 2016–17)

**DOI:** 10.1002/fsn3.3400

**Published:** 2023-05-06

**Authors:** Manuel L. Gaiser, Andrea S. Winkler, Stefanie J. Klug, Sandra Nkurunziza, Dominik Stelzle

**Affiliations:** ^1^ Chair of Epidemiology, Department of Sport and Health Sciences Technical University of Munich Munchen Germany; ^2^ Center for Global Health, Department of Neurology Technical University of Munich Munich Germany; ^3^ Department of Community Medicine and Global Health, Institute of Health and Society University of Oslo Oslo Norway; ^4^ Health Community Department, Faculty of Medicine University of Burundi Bujumbura Burundi

**Keywords:** Burundi, children under five, stunting, undernutrition

## Abstract

Burundi has one of the highest prevalence of stunting in the world. This study aimed to identify determinants of stunting among children under age five in Burundi. A total of 4993 children with anthropometric measurements from the 2016–2017 Burundi Demographic and Health Survey were included in the study. Stunting was assessed from the height‐for‐age *Z*‐scores (HAZ). Logistic regression models were analyzed to identify demographic, maternal, child‐related, and structural variables that influence stunting. In total, 56.9% of children under age five were stunted, of those 31.0% moderately and 25.9% severely. Multivariable logistic regression indicated that older children, male children (adjusted OR (aOR) = 1.41, 95% CI 1.24–1.61), and children who were perceived as small (aOR = 2.00, 95% CI 1.55–2.59) or very small at birth (aOR = 2.37, 95% CI 1.57–3.59) were significantly more likely to be stunted. Moreover, children of single mothers, with lower levels of education, who were underweight at the time of the survey (aOR = 1.95, 95% CI 1.42–2.68), who had short stature (aOR = 3.76, 95% CI 2.50–5.66) or who conceived more than four children (aOR = 1.22, 95% CI 1.05–1.42) were more commonly stunted. Stunting was more prevalent in rural areas (aOR = 2.53, 95% CI 1.72–3.73) and in households with no access to improved types of toilet facilities (aOR = 1.27, 95% CI 1.10–1.45). The results of this study show that the prevalence of stunting in children under age five in Burundi is alarmingly high and underscores the urgent need for decisive and determined action.

## Graphical abstract

The prevalence of stunting among children under age five in Burundi is among the highest in the world. The findings of our study on the determinants of stunting among children under age five in Burundi indicate that children, with male gender and who were perceived as small or very small at birth were significantly more likely to be stunted. Moreover, children of single mothers, with lower levels of education, underweight at the time of the survey, with short stature or who conceived more than four children were more commonly stunted. Stunting was more prevalent in rural areas and in households with no access to improved types of toilet facilities.

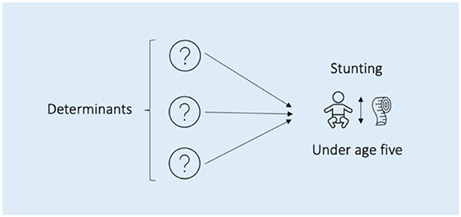

## 1 INTRODUCTION

In 2019, the World Health Organization (WHO) estimated that approximately 144 million children under age five were stunted (low height for age), representing 21.3% of all children in this age group globally (UNICEF et al., 2020). Of these children, 94% lived in either Asia (54%) or Africa (40%). The number of stunted children under age five has been significantly reduced since the year 2000 in nearly all WHO Regions, including Asia where prevalence nearly halved. In Africa, however, the number of stunted children has increased by more than 15% over the same period (UNICEF et al., 2020). Prevalence of stunting was estimated to be 35% of children under age five in sub‐Saharan Africa, while the eastern African region had with 37% the highest prevalence (Takele et al., 2022).

According to the Nutrition Landscape Information System of the WHO, stunting “reflects the cumulative effects of undernutrition and infections since and even before birth” (WHO, 2010). It is defined as a height for age below −2 times the standard deviation (SD) from the median of the WHO Child Growth Standard reference population (WHO, 2010).

Stunting is caused by a variety of interrelated factors that facilitate persistent or reoccurring disease and/or insufficient dietary intake (Akombi, Agho, Hall, Wali, et al., 2017; Black et al., 2013; UNICEF, 2015). The “UNICEF Conceptual Framework of the Determinants of Child Undernutrition” puts the conglomerate of facilitating factors in a context and differentiates between underlying and basic causes (UNICEF, 2015). Underlying causes include household food insecurity, inadequate care and feeding practices, unhealthy household environments, and inadequate health services. The basic causes are subdivided into (i) socio‐cultural, economic, and political context, (ii) inadequate financial, human, physical, and social capital, and (iii) the household access to adequate quantity and quality of resources, including land, education, employment, income, and technology (UNICEF, 2015).

In low‐income and middle‐income countries, infectious diseases such as diarrheal‐, respiratory‐, vector‐borne, and fever‐inducing diseases, as well as chronic diseases including heart, renal, and neurological diseases play a decisive role in the emergence of undernutrition, as they have a negative impact on how nutrients are digested, absorbed, transported, and utilized (Batte et al., 2017; Black et al., 2008, 2013; Silverstein, 2018; UNICEF, 2015).

A sufficient supply of nutrients during pregnancy and the first 1000 days of a child's life are of crucial importance for the healthy development of a child's brain and its physical growth (Black et al., 2013; UNICEF, 2015). Acute and chronic episodes of undernutrition in this period increase the risk for impaired cognitive, psychological, and physical development (Akombi, Agho, Hall, Wali, et al., 2017; Black et al., 2013; UNICEF, 2015; Victora et al., 2008).

In addition, stunting significantly increases the risk of mortality and morbidity (Black et al., 2013; UNICEF, 2015). Black et al. estimated that in 2008, 2.2 million deaths and 21% of the disability‐adjusted life years (DALYs) years were attributable to stunting, acute wasting, and intrauterine growth retardation among children under age five (Black et al., 2008).

Stunting in early childhood has long‐term effects that reach into adulthood. A systematic review found that undernutrition in early stages of life was significantly associated with shorter adult stature, fewer years of school education, lower income, lower offspring birthweight, and higher offspring perinatal mortality (Victora et al., 2008). Moreover, the review showed that children with episodes of childhood undernutrition had an elevated risk for increased blood pressure, fasting glucose levels, and blood concentration of low‐density fatty acids (Victora et al., 2008). It is assumed that children who were chronically or acutely undernourished during childhood have a significantly higher risk of being overweight in adulthood, which in turn, increases the risk of cardiovascular, oncological, and metabolic diseases (UNICEF, 2015; Victora et al., 2008). As these interdependencies demonstrate, high prevalence of stunting not only leads to devastating consequences for the affected children and their families but also hinders the sustainable economic and social development of countries.

Despite great efforts by the Burundian government and its development partners, prevalence of stunting of children under age five in Burundi is among the highest in the world (ENSNSAB, 2019). The prevalence only slightly decreased in the last decade from 58%, to 55.9% to 54.2% in 2010, 2016/2017, and 2019, respectively (ENSNSAB, 2019; ISTEEBU et al., 2012, 2017). The adverse effects of climate change and the return of more than 100,000 political refugees are expected to further aggravate the high level of food insecurity in Burundi in the future (UNHCR, UNDP, 2019). To be able to sustainably reduce the prevalence of stunting and to evaluate, prioritize, and implement future measures to combat undernutrition, scientific evidence on the factors associated with stunting is required.

The aim of this study is to identify the determinants of stunting of children under age five in Burundi and to provide key stakeholders, development actors, and policy makers with sufficient data to make informed decisions in their efforts to reduce the burden of stunting in Burundi.

## 2 METHODS

For this study, we analyzed data from the third Burundi Demographic and Health Survey 2016–2017 (MPBGP et al., 2016). The BDHS 2016–17 is a cross‐sectional population‐based household survey that assembled nationally representative data on various sociodemographic and health‐related parameters. It was conducted by the Institute of Statistics and Economic Studies of Burundi in collaboration with the National Institute of Public Health and with technical assistance from ICF International. The data collection phase was carried out between October 2016 and March 2017, and it was funded in a joint effort between the Burundian Government and its development partners.

In order to simplify statistical analysis, the collected data are provided in separate data files for different units of analysis. The data set analyzed in this study was derived from the Children Recode File (KR). This data file lists every child under age five that was born to interviewed woman within the selected households and contains general, socioeconomic, and health‐related information about the child, mother, and household. We constrained the analysis to children under age five (i) whose mothers lived in the household and could provide child‐related information, (ii) for whom anthropometric data were collected, and (iii) who were alive at the time of data collection.

### 2.1 Variables

#### 2.1.1 Dependent variables

The anthropometric indicator stunting was used to assess the nutritional status of the children. In the BDHS 2016–17, stunting was calculated by comparing the children's height with the median height of the WHO reference population of the same age. The deviance from the reference population is expressed in standard deviations and is referred to as a *Z*‐score. In this analysis, stunting was coded dichotomously (“stunted” and “not‐stunted”) according to the benchmark recommendations of the WHO Nutrition Landscape Information System (WHO, 2010). Children with *Z*‐scores below −2 SD from the median of the reference population were classified as stunted. A distinction was further made between moderately (between −2 SD and −3 SD) and severely stunted (below −3 SD) children. Anthropometric measurements of height and weight of children in the BDHS were carried out within randomly selected households. Weight was measured using the “SECA 878 flat” electronic scale. Size was measured using graded measuring rods (Shorr Board®). Children over 24 months were measured in an upright standing position, while infants under 24 months were measured in a reclining position.

#### 2.1.2 Included variables

Factors associated with undernutrition were selected on the basis of the “UNICEF Conceptual Framework of the Determinants of Child Undernutrition” from 2015 (UNICEF, 2015). A literature review on undernutrition studies (Akombi, Agho, Hall, Wali, et al., 2017; Amare et al., 2019; Ettyang & Sawe, 2016; Khan et al., 2019; Mukabutera et al., 2016; Poda et al., 2017) based on Demographic and Health Surveys (DHS) data served to detect appropriate indicator variables associated with stunting that were obtained within the BDHS 2016–17. Child‐related risk factors included the sex, age, birth order, perceived size at birth, the 14‐day prevalence of fever, diarrhea, and acute respiratory infection (ARI), breastfeeding status, place of birth, anemia status, and the number of children sleeping under an insecticide‐treated mosquito net. Maternal risk factors considered marital status, education, weight, stature, age at first birth, and the parity. On the household level, risk factors contained sex of household head, type of place of residence, region, and wealth quintile.

Almost all the selected independent variables from the BHDS 2016–17 were obtained from interviews with the children's mothers using ordinal or categorical survey scales. Exceptions were the determination of the anemia status that was assessed by drawing blood samples with self‐retractable lancets from children 6 months and older and analyzing it in a photometer (ISTEEBU et al., 2017). Children were considered anemic if they had hemoglobin levels <11.0 g/dL. If hemoglobin levels were <7.0 g/dL, children were severely anemic; between 7 and 11.0 g/dL, they were considered to be mildly/moderately anemic (Croft et al., 2018). Maternal height and weight were measured using the same anthropometric instruments to determine the height and weight of the children. The wealth index was constructed within the survey as a component measure that was derived from a number of different “easy‐to‐collect” proxy variables that reflect on the cumulative living standard of the household (Croft et al., 2018). Those proxy variables include information from household's ownership of a number of consumer items such as a car or television, dwelling characteristics such as flooring material, type of drinking water source, toilet facilities, and other characteristics that are related to wealth status (Croft et al., 2018). A detailed description of the variable names used from the BDHS 2016–17, their collection, response options, and the coding performed for the purpose of this study are shown in Appendix S1.

#### 2.1.3 Statistical analysis

The analyses were conducted using a weighted survey design to account for the two‐stage sampling design. The sample design applies a specific weighting factor to every child, according to its primary sampling unit and strata. The application of weights ensures nationally representative results. For the analyses, the R package ‘survey’ was used (Lumley, 2019). Prevalence estimates and percent distributions were calculated for all variables. Uni‐ and multivariable logistic regression models on stunting were performed. Factors that were statistically significant in univariable analyses were included in the multivariable logistic regression models. Variation inflation factors were analyzed to assess (multi‐)collinearity between independent variables. If two variables showed high collinearity, the more information‐revealing variable was kept. The other one was excluded from multivariable analyses. Anemia was excluded from multivariable analyses because of missing data. *p*‐values below .05 were considered significant. All statistical analyses conducted in this study were performed using R Studio 3.6.2.

## 3 RESULTS

### 3.1 Characteristics of the sample population

Within the framework of BDHS, a total of 16,637 households across 35 primary sampling units and 554 clusters from all 18 provinces were selected for the survey. At the time of survey, however, only 16,026 households were occupied. Another 29 households refused or did not take part in the survey for other reasons, resulting in a subset of 15,997 households that were interviewed successfully (99.7%). Of the 13,192 children under age five living in the interviewed households, anthropometric measurements of height and weight were taken from 6063 children. Due to incomplete data across the selected independent variables, an additional 1070 children had to be excluded from the analysis. A total of 4993 children under age five with complete data across all variables and information provided directly by the mother were included in the study.

The characteristics of the sample population are described in Table 1. All children were almost equally distributed among the five age groups and both sexes. The majority of children were born in second to fourth birth order (53.8%) and were perceived as large or average sized at birth (85.6%). Fever, diarrhea, or symptoms of acute respiratory infections (ARI) occurred among 41.0%, 22.1%, and 6.7% of children in the 2 weeks preceding the survey, according to their mothers. Almost all children were born in health facilities (87.1%) and had ever been or were still being breastfed (99.3%). Approximately half of the children (48.0%) did not sleep under insecticide‐treated mosquito nets and more than 60% had either moderate or severe anemia.

**TABLE 1:** Prevalence and percent distributions of the independent variables.

		*n*	Weighted *n*	%
		4993	5152	
*Child's characteristics*				
Child age	0–11 months	908	937	18.2
	12–23 months	999	1028	19.9
	24–35 months	962	985	19.1
	36–47 months	972	1030	20.0
	48–59 months	1152	1173	22.8
Child's sex	Male	2520	2594	50.4
	Female	2473	2558	49.6
Birth order	First	433	446	8.6
	2–4	2689	2772	53.8
	Fifth or higher	1871	1935	37.6
Size at birth	Very large	1788	1859	36.1
	Average	2477	2552	49.5
	Smaller than average	523	520	10.1
	Very small	205	222	4.3
Fever in the past 14 days		1950	2111	41.0
Diarrhea in the past 14 days		1069	1138	22.1
Acute respiratory infection in the past 14 days		334	345	6.7
Breastfeeding	Ever breastfed/still breastfed	4959	5115	99.3
	Never breastfed	34	37	0.7
Place of delivery	Home	645	662	12.9
	Health facility	4348	4490	87.1
Anemia (weighted *n* = 4733)	No anemia	1900	1880	39.7
	Mild/Moderate anemia	2540	2692	56.9
	Severe anemia	142	161	3.4
	Missing information	411	419	8.1
Slept under mosquito net (weighted *n* = 4992)	No, none	2381	2475	48.0
	Yes, some children	564	561	10.9
	Yes, all children	2048	2117	41.1
*Mother's characteristics*				
Marital status	Single	40	39	0.7
	Married/living with partner	4667	4806	93.3
	Separated/widowed/divorced	286	308	6.0
Education	No education	2411	2577	49.9
	Primary	2047	2123	41.1
	Secondary or higher	535	462	9.0
Work status	Not working	327	310	6.0
	Worked in the past year	261	290	5.6
	Currently working	4405	4552	88.4
Mother's weight	Underweight	745	769	14.9
	Normal	3767	3947	76.6
	Overweight	481	436	8.5
Mother's stature	Short stature	188	204	4.0
	Normal stature	4805	4948	96.0
Age at first birth	Under 18	799	835	16.2
	18–24	3530	3700	71.8
	25–49	664	618	12.0
Parity	One to four	2853	2941	57.1
	More than four	2140	2211	42.9
*Household characteristics*				
Sex of household head	Male	3979	4183	81.2
	Female	1014	969	18.8
Place of residence	Urban	773	453	8.8
	Rural	4220	4699	91.2
Region	Bubanza	299	280	5.4
	Bujumbura Rural	295	311	6.0
	Bururi	217	144	2.8
	Cankuzo	270	167	3.2
	Cibitoke	316	330	6.4
	Gitega	281	379	7.3
	Karuzi	319	304	5.9
	Kayanza	235	296	5.7
	Kirundo	303	387	7.5
	Makamba	296	319	6.2
	Muramvya	241	169	3.3
	Muyinga	342	441	8.6
	Mwaro	238	160	3.1
	Ngozi	287	400	7.8
	Rutana	283	242	4.7
	Ruyigi	289	283	5.5
	Bujumbura Mairie	193	240	4.6
	Rumonge	289	302	5.9
Wealth Index	Poorest	997	1080	21.0
	Poorer	965	1061	20.6
	Middle	1033	1117	21.7
	Richer	999	1046	20.3
	Richest	999	849	16.5
Source of drinking water	Improved water	4051	4174	81.0
	Unimproved water	942	979	19.0
Type of toilet facility	Improved toilet	2868	2730	53.0
	Unimproved toilet	2125	2423	47.0

Of the children's mothers, roughly 93% were living with a partner and almost half had no formal education. Approximately 15% of the mothers were underweight and about 4% had a stunted height (<150 cm). The majority of mothers (71.8%) conceived their first child between the age of 18 and 24 years and little over 40% of the mothers had given birth to more than four children.

The households of the included children were predominantly male headed (81.2%) and situated in rural areas (91.2%). Almost all households were equally distributed across the 18 provinces and the five wealth quintiles, except for a slight underrepresentation of the richest quintile. While 81.0% of the households had access to improved sources of drinking water, only half of the households had access to improved toilet facilities.

As illustrated in Table 2, the prevalence of stunting accounted for 56.9%, with 31.0% of the children being moderately and 25.9% being severely stunted.

**TABLE 2:** Prevalence and percent distributions of stunting in 5003 children in Burundi.

	*n*	Weighted *n*	%
Total	5003	5162	100
Not stunted	2218	2224	43.1
Stunted	2785	2938	56.9
Moderately stunted	1532	1600	31.0
Severely stunted	1253	1338	25.9

### 3.2 Determinants of stunting

Table 3 summarizes the results of the univariable and multivariable logistic regression models. In the univariable logistic regressions, several child‐, mother‐ and household‐related factors were significantly associated with stunting. These were the child's age, sex, perceived size at birth, place of delivery, and anemia status. Furthermore, all included mother‐related factors were associated with stunting, and of the household‐related factors place of residence, wealth quintile, source of water, and the type of toilet facility. All these factors apart from anemia (because of missing values) and wealth quintile (because of collinearity with mother's education) were included and adjusted for in the multivariable logistic regression model. Multivariable logistic regression indicated that children, in the four age groups from 12 to 59 months had significantly higher odds of being stunted compared to children between 0 and 11 months, with children in the age groups of 24–35 (adjusted OR (aOR) = 4.09, 95% CI 3.29–5.08) and 36–47 months (aOR = 4.48, 95% CI 3.59–5.59) showing the highest odds. Moreover, children with male sex (aOR = 1.41, 95% CI 1.24–1.61) and who were perceived as small (aOR = 2.00, 95% CI 1.55–2.59) or very small at birth (aOR = 2.37, 95% CI 1.57–3.59) were more likely to be stunted. Univariable logistic regression yielded a significant association for children with moderate (OR = 1.58, 95% CI 1.38–1.82) or severe anemia (OR = 3.46, 95% CI 2.25–5.33) and stunting. Since blood samples for anemia status have not been collected until 6 months of age, the variable anemia was not included in the multivariable models.

**TABLE 3:** Univariable and multivariable analysis analyses by stunting.

Variables		Univariable analysis (*n* = 5003)		Multivariable analysis (*n* = 5003)	
		OR	95% CI	aOR	95% CI
*Child's characteristics*					
Child age	0–11 months	1		1	
	12–23 months	2.91	2.34–3.62	3.09	2.47–3.87
	24–35 months	3.61	2.93–4.45	4.09	3.29–5.08
	36–47 months	4.01	3.25–4.95	4.48	3.59–5.59
	48–59 months	3.18	2.60–3.89	3.25	2.62–4.04
Child sex	Female	1		1	
	Male	1.31	1.16–1.49	1.41	1.24–1.61
Birth order	First	1		–	–
	2–4	0.83	0.66–1.03	–	–
	Fifth or higher	1.04	0.82–1.32	–	–
Size at birth	Very large	1		1	
	Average	1.22	1.05–1.41	1.25	1.07–1.46
	Smaller than average	1.84	1.44–2.36	2.00	1.55–2.59
	Very small	2.01	1.39–2.91	2.37	1.57–3.59
Fever	No fever	1		–	–
	Fever	1.12	0.97–1.28	–	–
Diarrhea	No diarrhea	1		–	–
	Diarrhea	1.01	0.88–1.17	–	–
Acute Respiratory Infection (ARI)	No ARI	1		–	–
	ARI	1.08	0.85–1.37	–	–
Breastfeeding	Ever breastfed/still breastfed	1		–	–
	Never breastfed	0.96	0.45–2.08	–	–
Place of delivery	Health facility	1		1	
	Home	1.47	1.22–1.77	1.16	0.96–1.40
Anemia^a^	No Anemia	1		–	–
	Mild/Moderate Anemia	1.59	1.38–1.83	–	–
	Severe Anemia	3.47	2.25–5.35	–	–
Slept under mosquito net	All children	1		1	
	Some children	1.19	0.96–1.48	1.14	0.90–1.43
	None	1.44	1.24–1.67	1.13	0.96–1.34
*Mother's characteristics*					
Marital status	Married/living with partner	1		1	
	Separated/widowed/divorced	1.49	1.10–2.01	1.36	0.99–1.86
	Never in union	2.36	1.09–5.14	3.21	1.13–9.13
Education	Secondary or higher	1		1	
	Primary	3.06	2.38–3.95	1.90	1.41–2.56
	No education	4.00	3.12–5.14	2.22	1.65–2.98
Work status	Not working	1		1	
	Working in the past year	1.97	1.25–3.10	1.31	0.81–2.11
	Currently working	2.03	1.45–2.84	1.47	1.02–2.12
Mother's weight	Overweight	1		1	
	Normal	2.49	1.98–3.12	1.71	1.34–2.18
	Underweight	3.21	2.34–4.40	1.95	1.42–2.68
Mother's size	Normal stature	1		1	
	Short stature	3.54	2.40–5.23	3.76	2.50–5.66
Age at first birth	25–49	1		1	
	18–24	1.37	1.07–1.74	0.98	0.78–1.24
	Under 18	1.58	1.18–2.10	1.16	0.87–1.54
Parity	One to four	1		1	
	More than four	1,33	1.16–1.51	1.22	1.05–1.42
*Household characteristics*					
Sex of household head	Male	1		–	–
	Female	0.90	0.75–1.08	–	–
Place of residence	Urban	1		1	
	Rural	4.11	2.89–5.81	2.53	1.72–3.73
Wealth index^b^	Richest	1		–	–
	Richer	2.28	1.75–2.98	–	–
	Middle	3.63	2.77–4.74	–	–
	Poorer	4.26	3.27–5.56	–	–
	Poorest	5.88	4.47–7.74	–	–
Source of drinking water	Improved water	1		1	
	Unimproved water	1.28	1.06–1.55	1.05	0.85–1.28
Type of toilet facility	Improved toilet	1		1	
	Unimproved toilet	1.52	1.33–1.73	1.27	1.10–1.45

^a^ Not included in multivariable analyses because of missing values.

^b^ Not included in multivariable analyses because of multicollinearity with mother's education.

Children of mothers who were never in union (aOR = 3.21, 95% CI 1.13–9.12), had primary (aOR = 1.90, 95% CI 1.41–2.56) or no education (aOR = 2.22, 95% CI 1.65–2.98), were currently working (aOR = 1.47, 95% CI 1.02–2.12), had normal weight (aOR = 1.71, 95% CI 1.34–2.18) or underweight (aOR = 1.95, 95% CI 1.42–2.68), had a short stature (aOR = 3.76, 95% CI 2.50–5.66), and conceived more than four children (aOR = 1.22, 95% CI 1.05–1.42) were more likely to be stunted.

Significant predictors for stunting on household level were children from rural areas (aOR = 2.53, 95% CI 1.72–3.73) and with no access to improved toilet facilities (aOR = 1.27, 95% CI 1.10–1.45).

### 3.3 Geospatial differences of stunting

**FIGURE 1:**
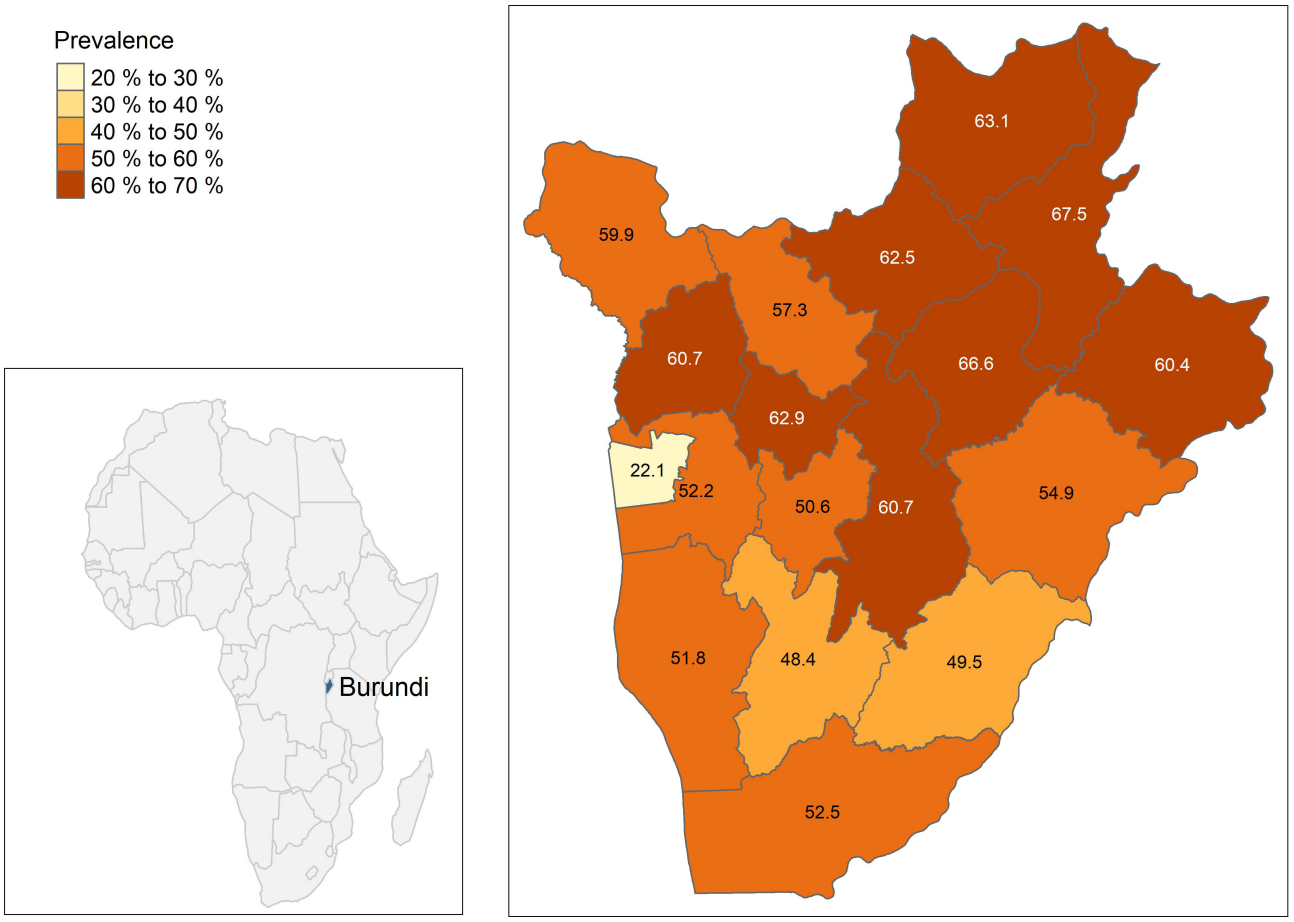
Prevalence of stunting by region among children.

Figure 1 displays the spatial differences in stunting across the 18 regions of Burundi. The map reveals that the prevalence of stunting was higher in the northern regions, especially in Karuzi (66.6%, 95% CI 60.4–72.7), Kirundo (63.1%, 95% CI 56.9–69.3), and Muyinga (67.5%, 95% CI 62.3–72.8), while the southern regions were less affected. Bujumbura Mairie had by far the lowest prevalence of stunting (22.1%, 95% CI 11.0–33.2) with 20%–40% lower rates compared to the other regions in the country.

## 4 DISCUSSION

In this study among children under age five in Burundi, we found a prevalence for stunting of 56.9%, with 31.0% of the children being moderately and 25.9% being severely stunted. Several child‐, mother‐, and household‐related factors were associated with stunting. Stunting was more common among boys, increased with age, peaking between 3 and 4 years, and was associated with small size at birth. Mothers who were underweight, short, single, or separated, and who had no, or little education were more likely to have stunted children. Generally, stunting was more common in rural areas, decreased with wealth of the household, and was more common in households with no access to improved toilet facilities.

Despite efforts to tackle undernutrition, the prevalence could not be reduced notably in the past few years and remains at an alarming level. Between 2010 and 2016–2017, the prevalence of stunting only decreased by 1.8%, and is considerably higher than in bordering countries like Tanzania, Rwanda, and the Democratic Republic of Congo with 34%, 38%, and 43%, respectively (Ministry of Health et al., 2016; MPSMRM et al., 2014; NISR et al., 2015). According to a meta‐analysis based on the most recent DHS data of 32 Sub‐Saharan African countries, Burundi even shows the highest prevalence of stunting in all of Sub‐Saharan Africa (SSA) (Akombi, Merom, Renzaho, & Hall, 2017).

We found several factors to be associated with the risk of stunting. Those include sociodemographic, maternal, child‐related, and structural factors.

### 4.1 Child factors associated with stunting

In our study, male children were significantly more likely to be stunted compared to female children. Similar results could be observed in a variety of studies across the globe, including a study in Burundi on the determinants of stunting among children between 6 and 23 months by Nkurunziza et al. (2017) (Akombi, Agho, Hall, Wali, et al., 2017; Kavosi et al., 2014; Khan et al., 2019; Ortiz et al., 2014). A systematic review that examined reasons for sex differences in stunting in SSA identified both social and biological reasons (Thurstans et al., 2020). Biologically, the higher risk of male children may be due to comparatively higher calorie requirements that originate from higher birth weight and a faster growth of male children in the early stages of life (Amare et al., 2019; Bork & Diallo, 2017; Thurstans et al., 2020). Reasons for the social origin of the sex differences could be the preferential treatment of female children that historically arises from a higher perceived value of the agricultural and domestic labor of women (Nkurunziza et al., 2017; Thurstans et al., 2020; Wamani et al., 2007).

Furthermore, we observed that children in the four age groups from 12 to 59 months had significantly higher odds of being stunted compared to children between 0 and 11 months of age. Similar results could be observed in several other studies around the globe and may be explained by the rise in calorie requirements, the insufficient transition from breastfeeding to complementary feeding practice, and the increased infection risk due to broader contact with the environment (Akombi, Agho, Hall, Wali, et al., 2017; Amare et al., 2019; Nkurunziza et al., 2017). In their prior study on stunting in Burundi, Nkurunziza et al. (2017) found that only 30% of all children between 18 and 23 received adequate complementary feeding indicating drastic deficiencies in the transitional phase.

In agreement with our findings, several other studies showed that children perceived as small or very small at birth had higher odds of being stunted compared to children who were perceived as very large (Akombi, Agho, Hall, Wali, et al., 2017; Khan et al., 2019; Nkurunziza et al., 2017). The maternal calorie and nutrient supply, as well as the maternal disease status during pregnancy, are thought to have a strong effect on the linear growth of children in the prenatal phase and may have irreversible effects on child growth potential after conception (Akombi, Agho, Hall, Wali, et al., 2017; Wali et al., 2020).

Furthermore, children with anemia, especially those who do not sleep under a mosquito net, were more commonly stunted which indicates that Malaria could be an important factor in stunting as well. According to the DHS Final Report 2016/17, the prevalence of malaria, similar to the prevalence of stunting, was highest in the northeastern regions (35%–51%) and lowest in the capital Bujumbura Mairie (3%) (ISTEEBU et al., 2017). The role of malaria in the emergence of anemia and stunting, however, should not be overestimated since prevalence of anemia was high in the capital Bujumbura Mairie (50%) even though prevalence of malaria was particularly low (ISTEEBU et al., 2017) and since there are several other factors that are associated with anemia (e.g., iron, vitamin B12, folic acid deficiency, other infectious diseases that cause anemia, etc.) (Chaparro & Suchdev, 2019).

### 4.2 Maternal factors associated with stunting

Maternal underweight was found to be a predictor for stunting in this analysis, as children of underweight mothers had significantly higher odds of being stunted compared to children of overweight mothers. Similar results could be observed in several studies based on DHS data across the globe (Akombi, Agho, Hall, Wali, et al., 2017; Khan et al., 2019; Poda et al., 2017). The high prevalence of stunting and maternal underweight is influenced by the high burden of household food insecurity that affected more than half of all households in Burundi in 2016 (World Bank Group, 2018). In the study by Nkurunziza et al. (2017), children who lived in households that were highly food insecure showed significantly higher odds of being stunted compared to children from food secure households. Reasons for the high prevalence of food insecurity in Burundi are the high dependency on the agricultural sector and insufficient agricultural productivity, which is in turn caused by insufficient agricultural knowledge and the lack of arable land and a high vulnerability toward adverse climate events such as droughts, floods, and land erosions (World Bank Group, 2018).

Children of mothers who were separated or never in union were significantly more likely to be stunted compared to children of mothers who were married or living with their partner. This association coincides with the findings of other studies, including the prior study on stunting in Burundi by Nkurunziza et al. (2017) (Abuya et al., 2012). The presence of two parents may increase the number of resources that can be directed toward the children and thus could result in higher income and possession, greater agricultural yield, and more time available for childcare.

We further observed that children of mothers with primary or no education had significantly higher odds of being stunted compared to children of mothers with secondary or higher education. Maternal educational attainment (and wealth) is highly intertwined with various factors that positively affect the nutritional status of both, mother and child through improvements in income, maternal self‐determination, dietary diversity and knowledge, hygiene awareness, child disease management, and access to healthcare and childcare services (Akombi, Agho, Hall, Wali, et al., 2017; Amare et al., 2019; Khan et al., 2019).

Elevated parity increased the odds of stunting in this analysis and is, moreover, associated with lower maternal education (Keats, 2018). Similar results could be observed in several cross‐sectional studies (Islam et al., 2013; Poda et al., 2017; Wong et al., 2014). With a growing number of children in a household, the risk of stunting accumulates, possibly because of the financial burden that is posed on households with an increasing number of children. These results support the current scientific understanding that a decrease in the prevalence of stunting can be accomplished through a reduction of maternal parity (Giroux, 2008).

### 4.3 Household factors associated with stunting

In Burundi, undernutrition is more common in rural areas. Similar results could be observed in several studies based on DHS data (Akombi, Agho, Hall, Merom, et al., 2017; Akombi, Agho, Hall, Wali, et al., 2017; Nkurunziza et al., 2017). However, there are also countries with contrary results such as Iran and Pakistan (Kavosi et al., 2014; Khan et al., 2019). In Burundi, the vast majority of people live in rural areas, most of which are densely populated. Thus, negative consequences of urbanization may be present in the rural areas as well. Households in urban areas, moreover, are less dependent on agriculture and tend to be employed in more economically profitable occupations (Bernoussi Marlène Kanga et al., 2013). This association may also be reflected by the low prevalence of stunting in Bujumbura Mairie which is mainly urban.

Regarding regional differences, prevalence of stunting was significantly higher in the northern, compared to the southern regions. This association could be caused by the higher population density in the northern regions (World Bank Group, 2018). Moreover, the southern regions of Burundi are less populated, at lower altitude, and flatter which is favorable for agriculture and competition for arable land. Furthermore, Lake Tanganyika in the southwest is a source for fishing and offers opportunities for irrigation of plants.

The importance of sustained food supply is also demonstrated by the fact that children are less often undernourished if they are living in wealthier households where food scarcity occurs less often. In the context of the DHS, the wealth index of a household is, in part, determined by the availability and type of a toilet facility. The lack of improved toilet facilities was associated with a higher risk for stunting in multiple studies in SSA and South Asia (Akombi, Agho, Hall, Wali, et al., 2017; Amare et al., 2019; Fink et al., 2011; Rah et al., 2015). The lack of improved toilet facilities increases the risk of children being exposed to fecal bacteria that can cause diarrheal diseases and intestinal worm infections and thus increases the susceptibility for stunting (Prüss‐Üstün, 2008; Rah et al., 2015).

### 4.4 Measures, outlook, and policy implications

The multitude of determinants observed in this study accentuates that stunting is caused by multiple factors and that a reduction can only be accomplished with multisectoral measures. In Burundi, structural factors such as low levels of education, life expectancy, and per capita income appear to have a major impact on the emergence of stunting. The precarious humanitarian situation within the country is most clearly reflected by the Human Development Index, in which Burundi ranks 185th out of 189 (UNDP, 2019). As this number indicates, Burundi is still struggling to overcome the aftermath of the civil war that struck the country between 1992 and 1999 and the subsequent political turmoil (World Bank Group, 2018). Another important factor explaining the prevalence of stunting is the high level of household food insecurity that affects over half of the population (World Bank Group, 2018). The influence of household food insecurity is also reflected in the strong association of infant birth weight, maternal stature, and maternal weight on stunting. A sufficient supply of nutrients during the pregnancy and the first 1000 days of a child's life are of crucial importance for the manifestation of stunting (Akombi, Agho, Hall, Wali, et al., 2017; UNICEF, 1990, 2015). For a sustainable reduction of stunting, measures should, therefore, be introduced that support particularly disadvantaged households in this critical phase. While some progress has been made to reduce stunting, challenges in food security evolve and grow in parts due to, e.g., climate change (UNICEF, 2022). Solutions to these challenges require strong and sustained political leadership. The World Food Program and UNICEF are advocating for the achievement of Sustainable Development Goal 2 “End hunger, achieve food security and improved nutrition, and promote sustainable agriculture” (UNICEF, 2022; United Nations, 2016; World Food Programme, 2019). In the latest report, UNICEF promotes reduction of cost for nutrition and calls governments, among other things, to support local small‐scale producers, and to secure their access to markets, to prioritize children's nutrition, and to embed nutrition in national social protection systems and investment strategies (FAO et al., 2020).

### 4.5 Strengths and weaknesses

This is the first study on the determinants of undernutrition among children under age five in Burundi based on the most recent Burundian Demographic and Health Survey from 2016/2017. The large sample size of close to 5000 children and the nearly perfect response rate of 99.7% are strengths of this study. Due to its two‐stage sample design, the results of the study can be interpreted as nationally representative. The generalized implementation of the DHS Program across the globe and the various studies further allow for an international comparison of the results.

A weakness of this study is the cross‐sectional study design, which neither allows for a causal interpretation of the study results nor takes into account seasonal or weather‐specific fluctuations (Amare et al., 2019). Due to the exclusion of all children with missing values, the results may have been distorted slightly. The exclusion of all children whose biological mothers could not be reached for the survey could have resulted in additional bias, as this could mean that particularly disadvantaged children were not included in the analysis (Giroux, 2008). A general problem with surveys is that they are prone to recall bias. This form of bias may not have had a large effect in this study, as most questions were answered by mothers and did not require long recall periods. Despite the questioning of the households in the national language Kirundo, incorrect information may have arisen due to misunderstandings in households that speak indigenous or tribal languages. Furthermore, measuring the height of children is also susceptible to measurement bias, particularly for children under 6 months of age where measurement was done in a reclining position (Amare et al., 2019). A further limitation of this study is that no information on dietary intake and household food insecurity was available. Future research is needed to address the influence of food insecurity and dietary intake on stunting.

## 5 CONCLUSION

The results of our analysis show that more than half of the children under age five in Burundi are affected by stunting. Despite great efforts by the Burundian government and its development partners, the burden of stunting has not been reduced significantly in the last decade. The many determinants for stunting observed in this study, including child, maternal, and household factors, accentuate that stunting is influenced in multifactorial ways. Thus, it is of crucial importance that measures consider the multifactorial origin of stunting. Our findings further suggest that stunting is significantly associated with variables that reflect the high food insecurity within the country.

## AUTHOR CONTRIBUTIONS

**Manuel L. Gaiser:** Conceptualization (lead); formal analysis (lead); methodology (lead); validation (supporting); visualization (supporting); writing – original draft (lead); writing – review and editing (supporting). **Stefanie J. Klug:** Investigation (supporting); project administration (supporting); supervision (supporting); validation (lead); writing – review and editing (supporting). **Dominik Stelzle:** Conceptualization (supporting); formal analysis (supporting); methodology (supporting); project administration (supporting); software (supporting); supervision (lead); validation (supporting); visualization (lead); writing – original draft (supporting); writing – review and editing (supporting). **Sandra Nkurunziza:** Formal analysis (supporting); investigation (supporting); methodology (supporting); validation (supporting); writing – review and editing (supporting). **Andrea S. Winkler:** Formal analysis (supporting); methodology (supporting); project administration (supporting); supervision (supporting); validation (supporting); writing – review and editing (supporting).

## CONFLICT OF INTEREST STATEMENT

All authors involved in this study declare no competing interests.

## ETHICS STATEMENT

Since this study is a secondary analysis, no additional ethical approval was required. Prior to the start of the BDHS 2016–17, ethical approval was obtained from the ICF Institutional Review Board and of the National Council of Statistical Information of Burundi.

## Supporting information


Appendix S1

## Data Availability

The data and protocol of the BDHS 2016–17 were retrieved from the DHS Program repository and are publicly available upon reasonable request: https://dhsprogram.com/data/dataset/Burundi_Standard‐DHS_2016.cfm?flag=1.
